# Detection of EXP1-Specific CD4+ T Cell Responses Directed Against a Broad Range of Epitopes Including Two Promiscuous MHC Class II Binders During Acute *Plasmodium falciparum* Malaria

**DOI:** 10.3389/fimmu.2019.03037

**Published:** 2020-01-22

**Authors:** Janna Heide, Nils H. Wildner, Christin Ackermann, Melanie Wittner, Matthias Marget, Alessandro Sette, John Sidney, Thomas Jacobs, Julian Schulze zur Wiesch

**Affiliations:** ^1^Infectious Diseases Unit, I. Department of Medicine, University Medical Center Hamburg-Eppendorf, Hamburg, Germany; ^2^German Center for Infection Research (DZIF), Partner Site Hamburg-Lübeck-Borstel-Riems, Hamburg, Germany; ^3^Department of Transfusion Medicine, University Medical Center Hamburg-Eppendorf, Hamburg, Germany; ^4^Division of Vaccine Discovery, La Jolla Institute for Immunology, La Jolla, CA, United States; ^5^Division of Infectious Diseases, Department of Medicine, University of California, San Diego, La Jolla, CA, United States; ^6^Protozoa Immunology, Bernhard-Nocht-Institute for Tropical Medicine, Hamburg, Germany

**Keywords:** malaria, *Plasmodium falciparum*, *Plasmodium vivax*, CD4+, CD8+, T cell epitope, HLA binding, HLA class II

## Abstract

**Background:** T cells are thought to play a major role in conferring immunity against malaria. This study aimed to comprehensively define the breadth and specificity of the *Plasmodium falciparum (P. falciparum)*-specific CD4+ T cell response directed against the exported protein 1 (EXP1) in a cohort of patients diagnosed with acute malaria.

**Methods:** Peripheral blood mononuclear cells of 44 patients acutely infected with *P. falciparum*, and of one patient infected with *P. vivax*, were stimulated and cultured *in vitro* with an overlapping set of 31 *P. falciparum*-specific 13-17-mer peptides covering the entire EXP1 sequence. EXP1-specific T cell responses were analyzed by ELISPOT and intracellular cytokine staining for interferon-γ production after re-stimulation with individual peptides. For further characterization of the epitopes, *in silico* and *in vitro* human leukocyte antigen (HLA) binding studies and fine mapping assays were performed.

**Results:** We detected one or more EXP1-specific CD4+ T cell responses (mean: 1.09, range 0–5) in 47% (21/45) of our patients. Responses were directed against 15 of the 31 EXP1 peptides. Peptides EXP1-P13 (aa60-74) and P15 (aa70-85) were detected by 18% (*n* = 8) and 27% (*n* = 12) of the 45 patients screened. The optimal length, as well as the corresponding most likely HLA-restriction, of each of these two peptides was assessed. Interestingly, we also identified one CD4+ T cell response against peptide EXP1-P15 in a patient who was infected with *P. vivax* but not *falciparum*.

**Conclusions:** This first detailed characterization of novel EXP1-specific T cell epitopes provides important information for future analysis with major histocompatibility complex-multimer technology as well as for immunomonitoring and vaccine design.

## Introduction

219 million cases of malaria and 435,000 deaths were recorded in 2017, mostly in Africa, and half of the world's population is at risk of the disease (1). *Plasmodium falciparum* (*P. falciparum*) is the species that causes the majority of deaths, and drug and insecticide resistance is increasing. A consistent solution would be the broad employment of a -thus far elusive- effective and durable protective malaria vaccine (2).

The exact mechanisms that lead to protection against malaria have not been fully defined, but both specific CD4+ and CD8+ T cells are thought to play a major role in conferring immunity against malaria (3–6). Obstacles in vaccine development are the large genome of *P. falciparum*, that encodes for more than 5,000 proteins (7). These malaria proteins exhibit great diversity and are partially only expressed at certain stages of the *plasmodial* life cycle, making them difficult targets for the immune system and vaccine design (8).

As a consequence, only relatively few *P. falciparum*-specific T cell epitopes located on a small number of malaria antigens have been identified and characterized in detail (9). Additionally, studies on the phenotype and function of *P. falciparum*-specific T cells are largely missing. Furthermore, detailed immunological studies that assess the T cell repertoire in correlation with patient characteristics [such as the origin and the human leukocyte antigen (HLA) alleles] and the clinical course of the disease [complicated vs. uncomplicated course, anemia, parasitaemia, or the C-reactive protein (CRP)] are lacking. Thus, fine mapping of the optimal length of T cell epitopes and investigation of their HLA-restriction will allow the synthesis of suitable major histocompatibility complex (MHC)-multimers for phenotypical analysis that are necessary to understand the function of T cells in malaria (10).

The 162 amino acid long exported protein 1 (EXP1) plays a pivotal role for the parasite at different stages of the life cycle. It is, for example, a component within the parasitophorous vacuole membrane (PVM) that separates and protects the parasite from the cytosol of the host cell (11). The sequence of EXP1 is well conserved across different strains of *Plasmodium* species (Supplementary Table 1). The expression of EXP1 during the liver and the blood stage of the *plasmodial* life cycle inside the human host potentially makes it an ideal target for *P. falciparum*-specific CD4+ and CD8+ T effector cells. CD8+ T cells are thought to play an important role during the liver stage because they are able to attack infected hepatocytes (that present peptides on MHC class I molecules) (3). CD4+ T cells are considered to mainly confer immunity during the blood stage in which erythrocytes (that do not carry MHC class I molecules) are being invaded by the parasite (12).

Former studies have shown that antibodies against EXP1 were able to inhibit parasite growth *in vitro* as well as *in vivo* (13, 14). Also, DNA vaccines containing the *P. falciparum* EXP1 molecule and synthetic peptides from the C-terminal region of EXP1 conferred protection in mice (15, 16).

EXP1 is expressed at two different life cycle stages where it is assumed to have important physiological functions (17), and it has shown encouraging results in previous vaccine studies. This makes EXP1 a promising target antigen for vaccine development (18–20). So far, only four CD4+ and six CD8+ EXP1-specific T cell epitopes have been described (Supplementary Figure 1) (21–25).

In this study we comprehensively defined 15 different *P. falciparum*-specific EXP1 CD4+ T cell epitopes using a previously established *in vitro* culture protocol for pathogen-specific T cells (26–29), and evaluated clinical parameters as well as patients' characteristics. We also performed fine mapping and HLA binding experiments that will allow the design of suitable MHC-multimers to characterize the phenotype of the T cell response in future studies (29).

## Materials and Methods

### Patient Cohort

PBMCs (peripheral blood mononuclear cells) of *P. falciparum* infected patients (*n* = 44) and one patient infected with *Plasmodium vivax*, as well as uninfected healthy controls (*n* = 10) collected at University Medical Center Hamburg–Eppendorf, were either used freshly or stored in liquid nitrogen (−196°C). *P. falciparum* infection was verified microscopically by experienced lab technicians in the diagnostic department of the Bernhard-Nocht-Institute for Tropical Medicine, Hamburg, Germany. Thick and thin blood smears were stained with 4% Giemsa and examined under oil immersion (original magnification ×100). Patients were further sub-stratified into two groups for some of the analysis. Group 1 (*n* = 11) included patients originally from Germany and group 2 (*n* = 31) included patients who were born in Africa. Two patients came from the Philippines and Jamaica. We also compared patients with a complicated (30) (*n* = 11) and uncomplicated (*n* = 33) course of the disease and we distinguished between patients that had the first malaria infection (*n* = 17) and patients that have had prior malaria infections (*n* = 27). Other clinical parameters including parasitaemia, CRP, hemoglobin and thrombocytes were also assessed and are shown in Table 1 (clinical details for patient HH-43 are unknown).

**Table 1:** Clinical and immunological patient characteristics.

**Patient**	**Age/Sex**	**Days since start of symptoms/therapy**	**Initial parasitaemia [%]**	**Complicated malaria**	**Prophylaxis**	**Number of malaria episodes**	**Country visited/Country of origin**	**CRP [mg/dl]**	**Hemoglobin [g/dl]**	**Thrombocytes [1000/μl]**	**Treatment regimen**	**Number of EXP1-specific CD4+ T cell responses**
HH-01	w 21	2/1	<1	No	No	>1	Togo/Germany	20	11.6	144	A/P	0
HH-02	m 37	6/2	<1	No	No	>1	Benin/Togo	114	12.6	102	PT/DA	0
HH-03	m 32	28/28	4.4	Yes	No	1	Angola, Cameroon, Senegal/Philippines	9	7.6	404	Artesunate + A/P	2
HH-04	m 47	5/3	8	Yes	No	1	Nigeria/Germany	107	15.0	32	Artesunate + A/P	0
HH-05	m 60	9/3	1	Yes	No	>1	Congo/Congo	108	9.5	7.4	PT/DA	0
HH-06	w 29	8/3	11	Yes	No	1	occupational: needlestick/Germany	67	7.7	118	Artesunate	2
HH-07	w 27	8/6	<1	No	No	1	Togo/Germany	14	13.9	68	PT/DA	3
HH-08	m 36	18/13	<1	No	No	1	Togo/Togo	<5	12.8	253	PT/DA	0
HH-09	w 27	10/3	<1	No	No	1	Ivory Coast, Togo/Ivory Coast	40	11.1	127	A/P	3
HH-10	m 25	20/2	<1	No	No	1	Guinea-Bissau/Guinea-Bissau	139	13.9	81	PT/DA	5
HH-11	m 40	10/1	7	Yes	No	1	Uganda/Jamaica	233	11.0	20	Artesunate	0
HH-12	m 56	7/3	>10	Yes	Yes (herbal)	1	Madagascar/Germany	82	12.6	130	Artesunate + A/P	0
HH-13	m 42	7/2	<1	No	No	1	Nigeria/Nigeria	225	12.2	61	PT/DA	0
HH-14	m 53	7/1	<1	No	Unregularly	1	Ivory Coast/Ivory Coast	73	13.7	128	A/P	1
HH-15	w 44	8/3	<1	No	No	>1	Cameroon/Cameroon	66	10.4	106	A/P	0
HH-16	m 52	6/3	<1	No	No	>1	Ghana/Togo	56	14.2	88	PT/DA	4
HH-17	m 63	9/0	<1	No	Unregularly	>1	Ghana/Ghana	138	12.6	53	PT/DA	0
HH-18	m 35	3/2	<1	No	Unregularly	>1	Nigeria/Nigeria	27	14.4	119	PT/DA	5
HH-19	m 19	5/2	<1	No	No	>1	Togo/Togo	<5	13.0	185	A/P	0
HH-20	m 52	6/2	1	No	No	>1	Ghana/Ghana	239	12.8	60	PT/DA	2
HH-21	m 31	8/2	<1	No	No	>1	Kenya/Germany	88	14.8	61	A/P	1
HH-22	w 43	5/3	<1	No	Unregularly	>1	Sierra Leone/Sierra Leone	87	11.7	82	PT/DA	2
HH-23	m 61	7/1	<1	No	Unregularly	>1	Cameroon/Cameroon	38	13.6	122	A/P	0
HH-24	w 35	6/2	<1	No	No	1	Nigeria/Germany	44	8.5	88	A/P	0
HH-25	m 41	5/1	<1	No	No	>1	Guinea/Guinea	167	13.9	72	PT/DA	3
HH-26	m 23	5/2	<1	No	No	1	Cambodia/Germany	66	13.2	51	A/P	1
HH-27	m 55	28/4	1	No	No	>1	Sudan/Sudan	46	10.9	141	PT/DA	1
HH-28	w 38	25/1	<1	No	No	>1	Ivory coast/Ivory Coast	102	13.1	244	A/P	0
HH-29	m 50	8/2	4	No	Yes (Lariam)	>1	Ghana/Ghana	223	12.1	50	PT/DA	4
HH-30	m 50	4/1	2	No	No	>1	Nigeria/Nigeria	109	12.7	86	PT/DA	0
HH-31	m 54	7/3	10	Yes	No	>1	Ghana/Ghana	77	11.3	125	PT/DA	2
HH-32	m 61	18/3	2	Yes	No	>1	Nigeria/Nigeria	9	10.4	168	Artesunate + A/P	0
HH-33	m 42	6/2	<1	No	No	>1	Nigeria/Nigeria	82	13.6	73	PT/DA	0
HH-34	m 72	4/2	7	Yes	No	>1	Nigeria/Nigeria	33	7.9	279	artesunate + A/P	0
HH-35	m 66	6/2	1	Yes	No	>1	Ghana/Ghana	14	11.7	116	Artesunate + A/P	0
HH-36	m 62	6/1	<1	No	No	1	Ghana/Ghana	76	15.7	71	A/P	0
HH-37	m 33	6/3	<1	No	No	>1	Benin/Benin	118	12.7	91	PT/DA	0
HH-38	m 58	13/1	<1	No	No	1	Ghana/Ghana	191	12.0	73	PT/DA	2
HH-39	m 36	3/2	<1	No	No	1	Ivory coast/Ivory Coast	87	12.7	107	PT/DA	2
HH-40	w 43	6/2	<1	No	No	>1	Togo/Togo	128	11.0	125	PT/DA	0
HH-41	w 49	5/4	<1	No	No	1	Nigeria/Germany	15	12.9	66	A/P	0
HH-42	m 43	4/2	<1	No	Unregularly	>1	Gabon/Germany	92	16.2	83	A/P	1
HH-43	w 26											0
HH-44	w 20	4/2	2	No	No	>1	Benin/Germany	88	11.9	104	A/P	1
HH-45	w 38	8/4	5	Yes	No	>1	Cameroon/Cameroon	171	7.1	134	Artesunate + A/P	2
**Average**	42.82	8.59/3.07	2.37					89.05	12.14	111.33		1.09
**Number**	45	44/44	44					44	44	44		45
**Standard deviation**	13.60	6.21/4.31	3.15					64.63	2.11	72.00		1.46
**Minimum**	19	2/0	<1					5.0	7.1	7.4		0
**Maximum**	72	28/28	11					239.0	16.2	404.0		5
**Median**	42	6.5/2	<1					82.0	12.6	96.5		0

*Patient HH-21 (marked in gray) was infected with P. vivax not with P. falciparum. All values refer to the first blood sampling. Patient HH-04 suffered from IgA nephritis as comorbidity, patient HH-08 and HH-31 from Schistosomiasis, patient HH-15 from HIV treated with highly active antiretroviral therapy (HAART), patient HH-23 from hepatitis C, patient HH-24 from Acinetobacter baumannii, patient HH-27 from hepatitis B and D, patient HH-33 from latent tuberculosis. A/P stands for treatment with atovaquon/ proguanil and PT/DA for piperaquine tetraphosphate and dihydroartemisinin*.

### EXP1 Peptides

Thirty-one 13-17-mer peptides overlapping by ten amino acids corresponding to the complete amino acid sequence of the EXP1 protein [Uniprot.org; UniProt ID: Q9U590 (31)] were synthesized (peptides&elephants, Hennigsdorf, Germany), and pooled into 3 pools of 10, 10 and 11 peptides (Table 2). For *in vitro* culture peptide pools were used at a total concentration of 10 μg/ml [concentration of a single peptide within the pool was accordingly 1 μg/ml (1/10)]. For the enzyme linked immunospot assays (ELISPOT) the concentration of single peptides was 10 μg/ml. Further peptides for truncation experiments and variants from different strains were also synthesized. Various sequences were found on uniprot.org (Supplementary Table 1) (32). We used the terms [EXP1; exported protein 1; circumsporozoite-related protein 1; circumsporozoite-related antigen 1] for the protein name and *P. falciparum* [PLAFA] or *P. vivax* [PLAVI] as organism term.

**Table 2:** Sequences of EXP1 peptides (*P. falciparum*, UniProt ID: Q9U590).

**Peptide**	**Peptide position**		**Amino acid sequence**																
EXP1-P01	1–15	Pool 1	M	K	I	L	S	V	F	F	L	A	L	F	F	I	I		
EXP1-P02	5–19		S	V	F	F	L	A	L	F	F	I	I	F	N	K	E		
EXP1-P03	10–24		A	L	F	F	I	I	F	N	K	E	S	L	A	E	K		
EXP1-P04	15–29		I	F	N	K	E	S	L	A	E	K	T	N	K	G	T		
EXP1-P05	20–34		S	L	A	E	K	T	N	K	G	T	G	S	G	V	S		
EXP1-P06	25–39		T	N	K	G	T	G	S	G	V	S	S	K	K	K	N		
EXP1-P07	30–44		G	S	G	V	S	S	K	K	K	N	K	K	G	S	G		
EXP1-P08	35–49		S	K	K	K	N	K	K	G	S	G	E	P	L	I	D		
EXP1-P09	40–54		K	K	G	S	G	E	P	L	I	D	V	H	D	L	I		
EXP1-P10	45–59		E	P	L	I	D	V	H	D	L	I	S	D	M	I	K		
EXP1-P11	50–64	Pool 2	V	H	D	L	I	S	D	M	I	K	K	E	E	E	L		
EXP1-P12	55–69		S	D	M	I	K	K	E	E	E	L	V	E	V	N	K		
EXP1-P13	60–74		K	E	E	E	L	V	E	V	N	K	R	K	S	K	Y		
EXP1-P14	65–79		V	E	V	N	K	R	K	S	K	Y	K	L	A	T	S		
EXP1-P15	70–85		R	K	S	K	Y	K	L	A	T	S	V	L	A	G	L	L	
EXP1-P16	75–89		K	L	A	T	S	V	L	A	G	L	L	G	V	V	S		
EXP1-P17	80–96		V	L	A	G	L	L	G	V	V	S	T	V	L	L	G	G	V
EXP1-P18	85–99		L	G	V	V	S	T	V	L	L	G	G	V	G	L	V		
EXP1-P19	90–104		T	V	L	L	G	G	V	G	L	V	L	Y	N	T	E		
EXP1-P20	95–109		G	V	G	L	V	L	Y	N	T	E	K	G	R	H	P		
EXP1-P21	100–114	Pool 3	L	Y	N	T	E	K	G	R	H	P	F	K	I	G	S		
EXP1-P22	105–119		K	G	R	H	P	F	K	I	G	S	S	D	P	A	D		
EXP1-P23	110–124		F	K	I	G	S	S	D	P	A	D	N	A	N	P	D		
EXP1-P24	115–129		S	D	P	A	D	N	A	N	P	D	A	D	S	E	S		
EXP1-P25	120–134		N	A	N	P	D	A	D	S	E	S	N	G	E	P	N		
EXP1-P26	125–139		A	D	S	E	S	N	G	E	P	N	A	D	P	Q	V		
EXP1-P27	130–144		N	G	E	P	N	A	D	P	Q	V	T	A	Q	D	V		
EXP1-P28	135–149		A	D	P	Q	V	T	A	Q	D	V	T	P	E	Q	P		
EXP1-P29	140–154		T	A	Q	D	V	T	P	E	Q	P	Q	G	D	D	N		
EXP1-P30	145–159		T	P	E	Q	P	Q	G	D	D	N	N	L	V	S	G		
EXP1-P31	150–162		Q	G	D	D	N	N	L	V	S	G	P	E	H				

*Peptide pool 1 consisted out of EXP1 peptides P1-10, pool 2 out of EXP1 peptides P11-20 and pool 3 out of EXP1 peptides P21-31. The entire EXP1 protein is 162 amino acids long and was divided into 31 overlapping peptides*.

### HLA Typing

High definition molecular HLA class I and II typing was performed at the Institute of Transfusion Medicine at University Medical Center Hamburg-Eppendorf, by polymerase chain reaction-sequence specific oligonucleotide (PCR-SSO) using the commercial kit SSO LabType as previously described (One Lambda, Canoga Park, CA, USA) (33).

### Bulk Stimulation of PBMCs

Cryopreserved PBMCs were thawed and cultivated at 1–5 × 10^6^ cells/ml for optimal results in 500 μl of R10 medium (RPMI 1640 medium with 10% FCS (Sigma Aldrich), 1% HEPES buffer and 1% Penicillin-Streptomycin). PBMCs were stimulated with EXP1 peptide pool 1 [EXP1 peptide 1-10 (EXP1-P01-10)], pool 2 (EXP1-P11-20) or pool 3 (EXP1-P21-31) (Table 2) at a final concentration of 10 μg/ml, together with 1 μg/ml of anti-CD28 and anti-CD49d antibodies [BD FastImmune™, clone: L293 (CD28) and clone: L25 (CD49d)] for 14 days. Medium with recombinant IL-2 (50 U/ml) was added when necessary. After 14 days, cells were re-stimulated with single EXP1-peptides (final concentration 10 μg/ml) and then assayed for interferon-γ (IFNγ) production by ELISPOT and intracellular cytokine staining (ICS) as previously described (28).

### ELISPOT Assay

ELISPOT assays were performed as previously described (26, 28, 34). We used 20,000 cells per well and responses were considered positive if the number of spots was at least three times the number of spots in the negative control. Single peptides were used at a concentration of 10 μg/ml. Anti-CD3-antibodies served as a positive control, DMSO and R10 as a negative control (35). All positive responses were confirmed by ICS assays following stimulation with the respective peptide.

### Intracellular IFNγ Staining and Flow Cytometry

**Figure 1:**
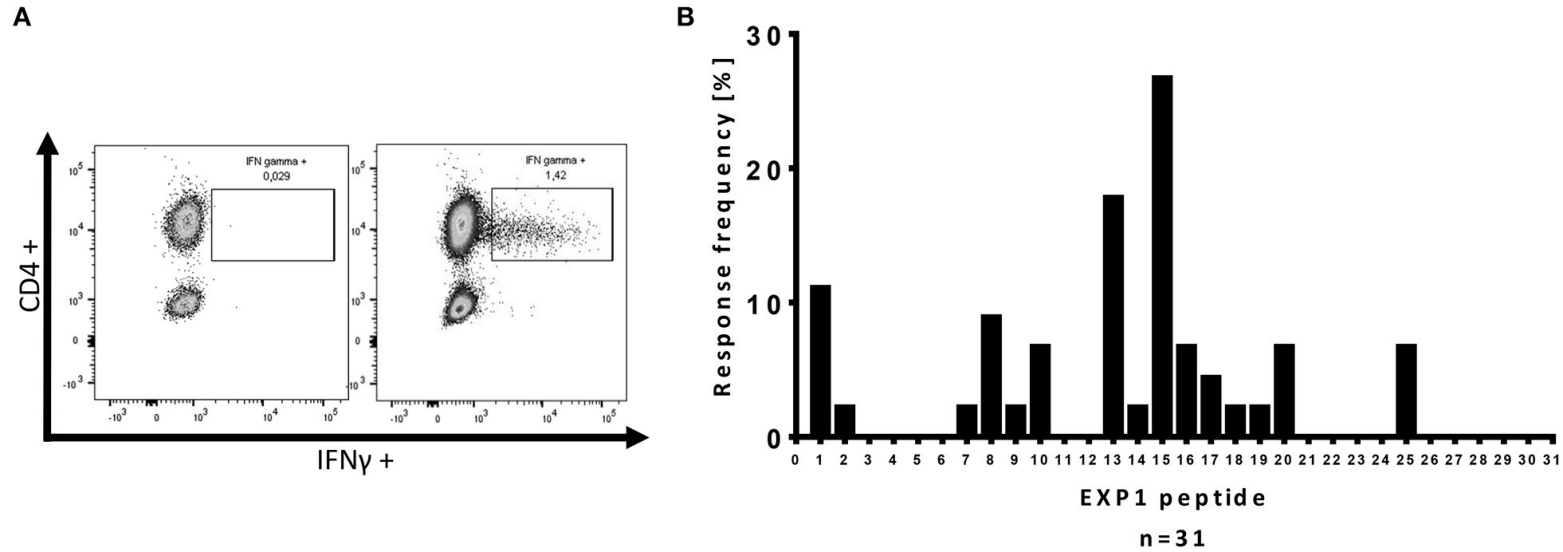
Intracellular cytokine staining (ICS) for IFNγ production of CD4+ T cells after re-stimulation with single EXP1 peptides. **(A)** Exemplary ICS dot blot of a *P. falciparum*-specific T cell response. Every positive response in the ELISPOT assay was confirmed by re-stimulation with a single peptide intracellular cytokine staining (ICS) assay for IFNγ production. CD4+ T cells were gated (an exemplary gating strategy is shown in Supplementary Figure 2), (left) negative control, (right) positive peptide response against EXP1-P15 by HH-03. DMSO and R10-medium were added to the negative control. **(B)** Overview of the relative location of the *P. falciparum*-specific T cell responses detected by one or more patients of the cohort against one or more of the 31 single EXP1 peptides. Response frequency (RF) is the number of patients that had a specific T cell response against a single peptide divided by the number of patients that were tested (*n* = 45). The center of EXP1, EXP1-P13 to EXP1-P20 (aa60-109), showed a high immunogenicity.

ICS was performed as previously described (26, 28). 5 × 10^5^ PBMCs were stimulated with the corresponding EXP1 peptide at a final concentration of 10 μg/ml before blocking the secretion with 10 μg/ml Brefeldin A (Sigma Aldrich) 1 h after stimulation. Cells were then incubated at 37°C overnight and stained with the Zombie NIR Fixable Viability kit for live cells as well as surface antibodies anti-CD3 (clone: Okt3; AlexaFluor 700), anti-CD4 (clone: SK3; PerCP-Cy5.5) and anti-CD8 (clone: RPA-T8; Brilliant Violet 786) (all antibodies by BioLegend). After fixation and permeabilization (eBioscience™, Foxp3/Transcription Factor Staining Buffer Set), cells were stained with anti-IFNγ-antibodies (clone: 4S.B3; PE- Texas red; BioLegend). Cells were then analyzed on a BD LSRFortessa (BD Bioscience). An exemplary gating strategy is shown in Supplementary Figure 2. We defined a T cell response as positive when the percentage of CD4+ T cells within the gate for IFNγ was three times higher than the negative control and the population could be clearly separated from the negative control (26, 28, 36). DMSO and R10 were added to the negative control. Figure 1A shows an exemplary ICS result of an EXP1-specific CD4+ T cell response.

### *In vitro* HLA Binding Assays and *in silico* Prediction

*In vitro* binding assays with 14 of the peptides that elicited a response were performed using purified HLA-DR molecules, as previously described (Table 3) (37). MHC class II *in silico* binding predictions were defined, using the IEDB analysis resource (38–40), combining predictions from the ANN (41, 42) and SMM (43) algorithms (Supplementary Table 2A).

**Table 3:** *In vitro* binding predictions of 14 *P. falciparum*-specific peptides to 17 frequent DRB1, DRB3, DRB4, and DRB5 types.

**Name**	**Sequence**	DRB1^*^ 01:01	DRB1^*^ 03:01	DRB1^*^ 04:01	DRB1^*^ 04:04	DRB1^*^ 04:05	DRB1^*^ 07:01	DRB1^*^ 08:02	DRB1^*^ 09:01	DRB1^*^ 10:01	DRB1^*^ 11:01	DRB1^*^ 13:01	DRB1^*^ 13:02	DRB1^*^ 15:01	DRB3^*^ 01:01	DRB3^*^ 02:02	DRB4^*^ 01:01	DRB5^*^ 01:01	**Alleles bound**	**Number of responses**
EXP1 peptide 1	MKILSVFFLALFFII	21,399	–	5,053	9,126	3,520	9,191	5,449	3,587	4,939	39,632	3,115	–	3,359	–	–	25,221	10,881	0	5
EXP1 peptide 2	SVFFLALFFIIFNKE	11,720	12,317	4,201	2,606	4,245	7,986	2,967	**648**	3,990	–	6,465	–	1,549	10,639	–	17,426	6,950	1	1
EXP1 peptide 7	GSGVSSKKKNKKGSG	–	–	–	–	–	–	–	–	–	–	1,982	–	–	–	–	–	4,556	0	1
EXP1 peptide 8	SKKKNKKGSGEPLID	–	–	–	–	–	–	29,998	–	–	–	–	–	–	–	–	–	–	0	4
EXP1 peptide 10	EPLIDVHDLISDMIK	6,016	6,770	27,548	1,108	30,955	–	2,840	–	–	–	–	–	29,167	7,963	–	**162**	–	1	3
EXP1 peptide 13	KEEELVEVNKRKSKY	–	–	–	–	–	–	–	–	–	**142**	1,189	–	–	–	–	17,926	1,123	1	8
EXP1 peptide 14	VEVNKRKSKYKLATS	2,345	–	**58**	–	**83**	8,296	–	**77**	**295**	–	5,739	–	6,103	13,943	5836	–	**89**	5	1
EXP1 peptide 15	RKSKYKLATSVLAGLL	**1.1**	5,823	**1.2**	**48**	**7.4**	**1.4**	11,920	**3.9**	**1.8**	**7.0**	–	39043	**195**	**47**	**2.0**	**230**	**1.0**	13	12
EXP1 peptide 16	KLATSVLAGLLGVVS	**11**	10,820	**380**	**454**	**539**	**133**	1,152	1,364	**152**	**810**	32,443	–	2,270	6,098	**481**	**567**	**244**	10	3
EXP1 peptide 17	VLAGLLGVVSTVLLGGV	**23**	–	1,259	**47**	**824**	**4.6**	**49**	**12**	**418**	13,725	14,178	33431	**5.0**	28,468	14877	2,394	**238**	9	2
EXP1 peptide 18	LGVVSTVLLGGVGLV	**19**	–	2,052	**806**	5,017	**433**	1,813	**491**	1,893	6,627	8,557	3227	**300**	13,497	–	**194**	2,471	6	1
EXP1 peptide 19	TVLLGGVGLVLYNTE	**8.6**	–	2435	20,079	4,947	3,951	–	4,452	33,954	–	7,440	–	**219**	–	–	8,277	1,180	2	1
EXP1 peptide 20	GVGLVLYNTEKGRHP	8,937	4,491	**26**	**9.1**	2,951	–	**9.3**	–	7,971	**183**	**122**	17121	**24**	–	4993	**81**	**539**	8	3
EXP1 peptide 25	NANPDADSESNGEPN	19,573	–	16,699	12,500	18,213	–	4,012	–	33,606	23,222	–	–	1,097	–	–	12,018	31,109	0	3

*The binding capacity is displayed as IC50 value deduced from in vitro experiments with selected purified HLA-DR molecules and overlapping EXP1 peptides. High affinity binding is defined as IC50 <1,000 nM and highlighted by bold font. For reasons of comprehensibility, values larger than 40.000 nM are indicated by a dash. The total number of alleles bound as well as the number of CD4+ T cell responses against the responding peptide is shown. EXP1-P15 bound to the highest number of DR-molecules (13 different alleles) in vitro*.

### Statistical Analysis

All flow cytometric data were analyzed using FlowJo 10.5.0 software (Treestar, Ashland, OR, USA). Statistical analyses were carried out using the Prism 7.0 software (GraphPad software, San Diego, CA). Mann Whitney test or the Kruskal–Wallis test with Dunn's post-test was performed throughout all samples for inter group comparisons. Spearman's correlation was performed for bivariate correlation analyses. Data are expressed as means with standard deviations (SD) or with standard error of mean (SEM). *P*-values less than or equal to 0.05 were considered significant.

## Results

### Clinical Features of the Study Cohort

The clinical data of all patients as well as other characteristics are summarized in Table 1. The clinical cohort consisted of 45 malaria patients of which 44 patients were infected with *P. falciparum;* one patient was infected with *P. vivax* (marked in gray). 13 patients were female (29%) and 32 were male (71%), and the average age was 42.8 years (range: 19–72 years). Eleven patients (24.4%) were born in Germany, 31 patients (68.9%) originated from the African continent, two patients were from the Philippines and Jamaica. The vast majority of patients who were treated for malaria at University Medical Center Hamburg-Eppendorf had traveled and returned from Western Africa: nine patients had traveled to Nigeria, eight patients to Ghana and five patients to Togo (Supplementary Figure 3). Patient HH-21 who was infected by *P. vivax* had traveled to Kenya, where growing evidence of *P. vivax* infection could be shown (44). *P. falciparum* infection was excluded by PCR and the patient had no medical record of a previous *P. falciparum* infection. Only one patient had traveled outside of Africa (to Cambodia). Eleven patients had a course of disease defined as complicated, while 34 courses of the disease were defined as uncomplicated. Further clinical parameters are shown in Table 1.

Initial parasitaemia was on average 2.37% (range: <1–11%), CRP 89 mg/dl (range: 5–239 mg/dl), hemoglobin 12.1 g/dl (range: 7.1–16.2 g/dl), and thrombocytes 111.300 /μl (range: 7,400–404,000 /μl). The average number of days between the start of symptoms and the blood sampling for our study was ~8.6 (range: 2–28 days). The number of days between the start of therapy and the blood sampling for our study was about 3.1 (range: 0–28 days). Patient HH-17 had a blood draw before therapy for malaria was initiated.

### Detection of EXP1-Specific T Cell Responses After *in vitro* Cultivation With IL-2

The *P. falciparum*-specific T cell response has so far not been comprehensively assessed for neither the majority of *P. falciparum*-specific antigens in general, nor for EXP1 in particular (9). In order to evaluate the EXP1-specific T cell response on an epitope level, we first looked at the *ex vivo* IFNγ production of PBMCs of acutely infected malaria patients after stimulation with single peptides of an overlapping 13-17-mer peptide set covering the entire *P. falciparum* antigen EXP1. Most samples were drawn 1-4 days after admission to the hospital (average 3.07 days) shortly before, during or after initiation of anti-malaria therapy, only 3 samples were taken at later timepoints.

However, we detected only one T cell response with the *ex vivo* ELISPOT in a total of ten patients (data not shown). The fresh blood sample of patient HH-38 showed an *ex vivo* response against EXP1-P15. After the *in vitro* culture, the same patient responded again to EXP1-P15 and additionally to EXP1-P10. These results are in line with the results of previous studies that showed that the magnitude of the *ex vivo P. falciparum*-specific CD4+ and CD8+ T cell response is low (23, 45–47). No EXP1-specific T cell responses were detectable after 14 days of *in vitro* culture of 10 healthy controls that had never traveled to a malaria endemic area (data not shown).

Due to the presumably low *ex vivo* precursor frequencies of *P. falciparum*-specific T cells, we employed a previously used, ultra-sensitive, *in vitro* approach (26–28, 48). In short: our peptide set was divided into three different peptide pools (Table 2) and we started three parallel cell cultures per patient, stimulating them with each pool for 14 days (Pool 1: EXP1-P1-10, Pool 2: EXP1-P11-20, Pool 3: EXP1-P21-31). This was followed by a single peptide ELISPOT for IFNγ-production against re-stimulation with the individual peptides. The 31 overlapping single peptides covered the entire EXP1 protein, and PBMCs from 45 patients were tested. Every positive response in the ELISPOT assay was confirmed by re-stimulation with the according single peptide and subsequent intracellular cytokine staining (ICS) assay for IFNγ production. Figure 1A shows an exemplary ICS result of an EXP1-specific CD4+ T cell response.

With this approach, we were able to distinguish one or more responses directed against 15 of the 31 overlapping EXP1 peptide specificities (48.4%). We detected EXP1-specific CD4+ T cell responses in nearly half of our patients (21/45, 47%) but, surprisingly, only four patients showed a CD8+ T cell response (4/45, 8.9%) (Supplementary Figure 4B). Patients recognized an average of 1.09 CD4+ restricted peptides (range 0–5), with a total number of 49 CD4+ T cell responses in 45 patients. The average frequency of IFNγ+CD4+ T cells was 1.12% (range: 0.2–4.84%) (data not shown). Figure 1B gives an overview of the distribution of the different peptide-specific T cell responses. Most responses were directed against epitopes that are located in the center of the EXP1 protein. Eight patients (response frequency (RF): 17.8%; 8/45) showed a CD4+ T cell response against EXP1-P13 (aa60-74), and 12 patients responded to EXP1-P15 (aa70-85) (RF: 26.7%, 12/45). Interestingly, the patient who was infected with *P. vivax* also responded to EXP1-P15. The sequences of *P. falciparum* EXP1-P15 (**RKSKYKLATSVLAGLL**) and *P. vivax* EXP1 aa68-82 (**KKSNYKLATTVLASAL**) were then compared and show a high degree of sequence homology (Supplementary Table 1).

We also longitudinally analyzed, in selected patients, the EXP1-specific T cell responses in the long-term follow-up. The patient HH-06 showed a large response against EXP1-P15 (frequency: 1.86% IFNγ+CD4+) and EXP1-P25 (frequency: 1.88% IFNγ+CD4+) during acute infection. However, 12 months after the first sampling a specific T cell response could not be detected from a cell culture started directly with fresh blood (data not shown). The patient had stayed in Germany and no further malaria infections had occurred in the intervening 12 months. By contrast, in patient HH-27, who responded to EXP1-P16 (frequency: 3.91% IFNγ+CD4+), we could detect responses against EXP1-P16 (frequency: 9.73% IFNγ+CD4+) and EXP1-P10 (frequency: 0.25% IFNγ+CD4+) in a sample collected 7 years prior to the present assays. During these 7 years, several subsequent malaria infections had occurred as reported by the patient and patient's health provider.

Of the 45 patient samples utilized, seven samples were cultured from fresh samples, of which three patients showed a CD4+ T cell response (42.9%, 3/7). The other 38 samples were stored in liquid nitrogen before analysis, and of these 18 patients showed a T cell response (47.4%, 18/38). Thus, there were no significant differences in the breadth of the detected responses as a function of sample storage conditions (data not shown).

### The Breadth of the EXP1-Specific CD4+ T Cell Response Does Not Correlate With the Clinical Course of Acute Malaria

**Figure 2:**
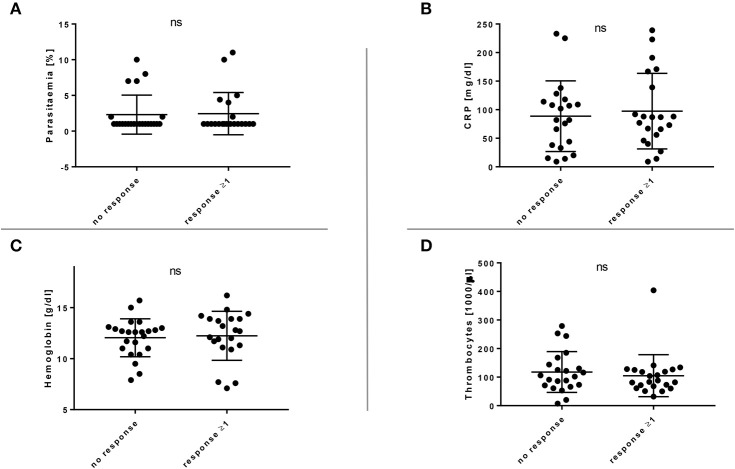
**(A–D)** Breadth of the EXP1-specific T cell response in comparison with relevant clinical parameters. Clinical parameters, including parasitaemia, CRP, hemoglobin and thrombocytes were examined but no significant (ns) correlation with the CD4+ T cell response could be shown. **(A)** Response or no response compared to parasitaemia [%]. **(B)** Response or no response compared to C-reactive protein (CRP) [mg/dl]. **(C)** Response or no response compared to hemoglobin [g/dl]. **(D)** Response or no response compared to thrombocytes [1,000/μl].

Several clinical parameters, including parasitaemia, CRP, hemoglobin, and thrombocyte count, were examined, but no significant correlation between the number of T cell responses and any of the parameters could be identified (Figure 2). Similarly, no correlation was found between age or gender and the CD4+ T cell response (Supplementary Figure 5). The patients' origin, the duration of the malaria therapy and the clinical course of the disease (uncomplicated and complicated) did also not correlate with the breadth of the CD4+ T cell response (Supplementary Figures 6A–C). When comparing the number of *in vitro* responses between different patient groups, we could not see significant differences between patients with their first episode of diagnosed malaria infection (52.9%, 9/17) vs. patients who had a documented history of a past malaria infection (44.4%, 12/27) (Supplementary Figure 6D).

### HLA Binding and Restriction Experiments

Table 4 lists the 45 donors examined in the present study along with their HLA-DRB1^*^ typing data and summarizes the individual patient-specific EXP1-peptide response patterns. The finding that certain peptides were recognized by a large proportion of subjects with acute *P. falciparum* infection, despite the fact that they expressed different HLA class II molecules, let us hypothesize that these were comparatively promiscuous MHC class II binding EXP1-specific CD4+ T cell epitopes (i.e., peptides recognized in the context of multiple HLA class II molecules).

**Table 4:**
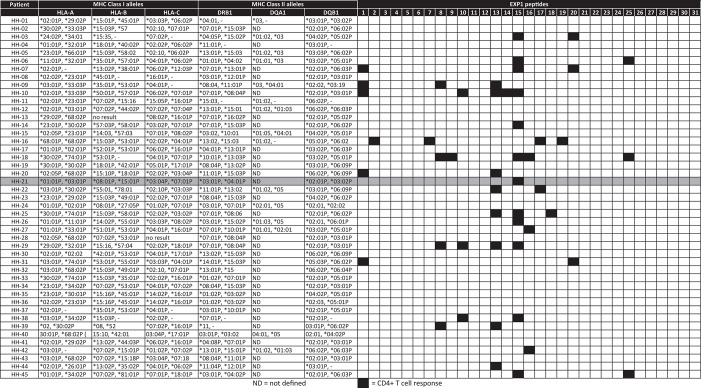
Overview of HLA type and corresponding EXP1-specific CD4+ T cell response.

*P. falciparum-specific CD4+ T cell response: black. Patient HH-21 (marked in gray) was infected with P. vivax not with P. falciparum. A total number of 49 CD4+ T cell responses was detected in 45 malaria patients with one or more EXP1-specific CD4+ T cell responses (mean: 1.09, range 0-5) in 47% (21/45) of our patients. Most responses were directed against EXP1-P15 (aa70-85) with a total number of 12 responses, followed by EXP1-P13 (aa60-74) that showed eight CD4+ T cell responses*.

To test this hypothesis, we measured the *in vitro* capacity of the most frequently recognized EXP1 peptides to bind to a panel of 17 of the most prevalent human HLA-DR molecules, since the majority of HLA class II restricted CD4+ T cell responses are presented by these molecules (49, 50). Peptides with an IC50 of 1,000 nM or lower, a threshold previously found associated with the vast majority of HLA class II-restricted T cell epitopes, were considered binders to the respective DR molecule (51–53). As shown in Table 3, in general the peptides located in the middle of the protein (i.e., EXP1-P14-18; aa65-99) were associated with the most promiscuous binding (range: 5-13 of the 17 DR tested). These include EXP1-P14 which bound to five, EXP1-P15 to 13, EXP1-P16 to 10 and EXP1-P17 to nine different HLA molecules (Table 3).

The most frequently recognized peptides were promiscuous in binding to multiple HLA-DR molecules, with each of the peptides binding between zero and 13 different DR molecules. Furthermore, some of these peptides were able to bind to several HLA-DR molecules with extremely high affinities (IC50 <10 nM) (Table 3). EXP1-P15, for example, showed excellent binding to 13 HLA molecules: DRB1^*^01:01 (1.1 nM), DRB1^*^04:01 (1.2 nM), DRB1^*^04:04 (48 nM), DRB1^*^04:05 (7.4 nM), DRB1^*^07:01 (1.4 nM), DRB1^*^09:01 (3.9 nM), DRB1^*^10:01 (1.8 nM), DRB1^*^11:01 (7.0 nM), DRB1^*^15:01 (195 nM), DRB3^*^01:01 (47 nM), DRB3^*^02:02 (2.0 nM), DRB4^*^01:01 (230 nM) and DRB5^*^01:01 (1.0 nM). This binding pattern is in line with the HLA molecules of the patients that responded to EXP1-P15. Peptides that bound to zero alleles in our DRB *in vitro* assay may be restricted by DQ or DP molecules.

Supplementary Table 2B shows the most likely HLA-restriction considering the *in vitro* binding data as well as the responders' HLA molecules. Nine out of 12 patients who recognized EXP1-P15 expressed at least one of the HLA molecules mentioned above. The most common HLA molecule expressed in patients of our cohort who recognized EXP1-P15 was DRB1^*^07:01 (six patients). Interestingly, six patients from our cohort also expressed DRB1^*^07:01 but did not recognize EXP1-P15. EXP1-P13 bound to DRB1^*^11:01 in the *in vitro* assay (142 nM). This is consistent with the fact that out of eight patients who recognized EXP1-P13, five were carrying the HLA-DRB1^*^11 molecule.

These results indicate that certain EXP1 peptides, especially those that are located in the center of the protein like EXP1-P15 are potentially promiscuous T cell epitopes.

### Fine Mapping of EXP1-P13 and P15

**Figure 3:**
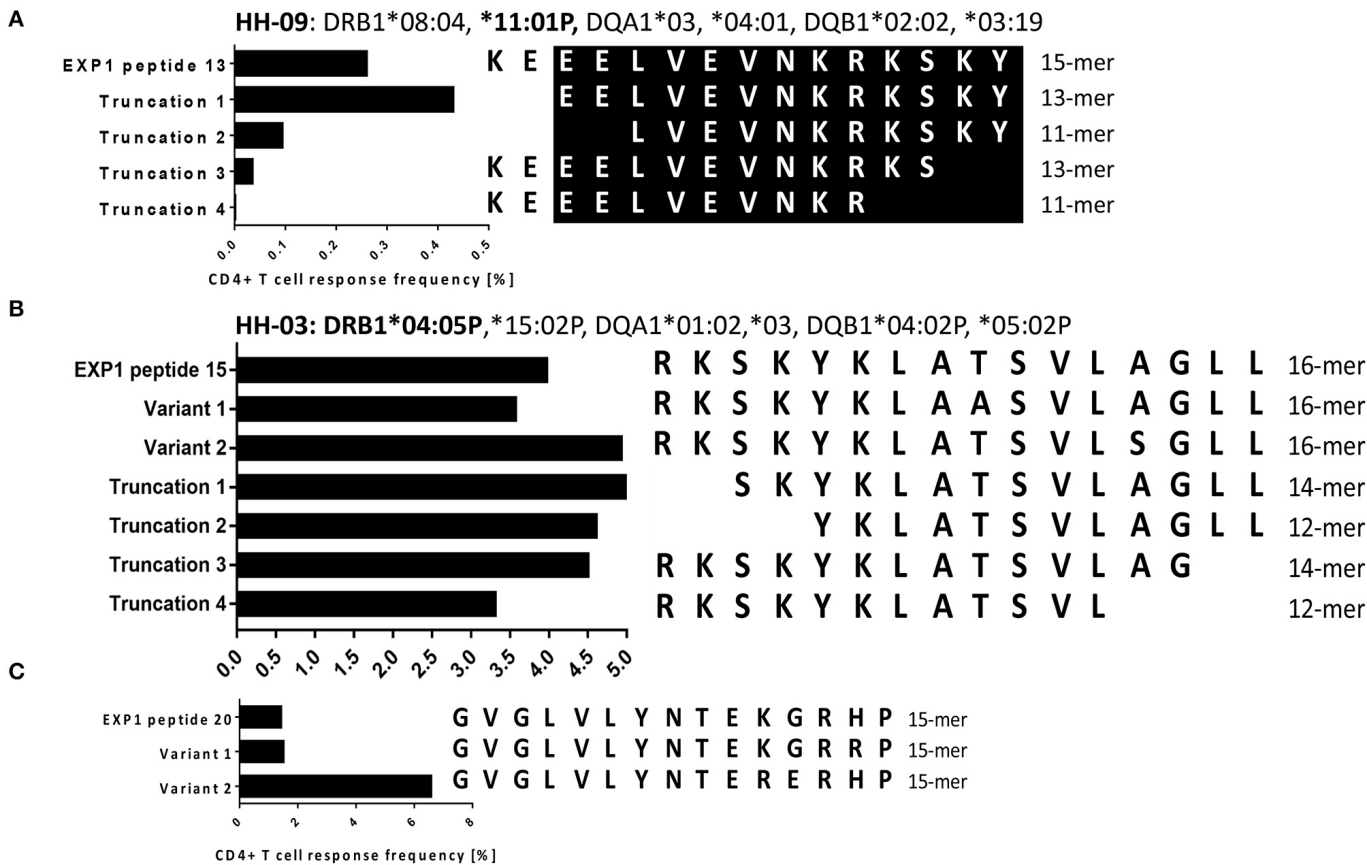
**(A)** Fine-mapping of malaria epitope EXP1-P13 (HH-09). **(B)** Fine-mapping of EXP1-P15 (HH-03). **(C)** Testing of variants for EXP1-P20 (HH-03). Experiments were performed by ICS for IFNγ production after stimulation with peptides of different length and with varying sequences. **(A)** For EXP1-P13 we used 15-, 13-, and 11-mer N- as well as C- terminal truncations with PBMCs of patient HH-09 (DRB1*08:04, *11:01P). Truncation 1 showed the highest CD4+ T cell response. The core epitope of EXP1-P13 is marked in black. **(B)** 16-, 14-, and 12-mer N- as well as C- terminal truncations were tested for EXP1-P15. PBMCs of patient HH-03 (DRB1*04:05P,*15:02P) were used for this experiment. EXP1-P15 Truncation 1 showed the highest CD4+ T cell response as well as EXP1-P15 Variant 2 which contains Serine (S) instead of Alanine (A). For EXP1-P15 and P20 variants were tested. Variant 1 of EXP1-P20 contained the amino acid Arginine (R) instead of Histidine (H) and Variant 2 Arginine (R) instead of Lysine (K).

To narrow down the minimal length of the epitopes within EXP1-P13 and P15, we tested the magnitude of the frequency of responding CD4+ T cells by the use of truncations (Figure 3). We first cultivated PBMCs of patients who had shown a strong response against the corresponding peptide with the original peptide (concentration 10 μg/ml) and then tested the magnitude of the IFNγ responses by ICS. The highest CD4+ T cell frequency for EXP1-P13 was reached by patient HH-09 by EXP1-P13 truncation 1 (13-mer truncation: **EELVEVNKRKSKY**). For EXP1-P15 the 14-mer EXP1-P15 truncation 1 (**SKYKLATSVLAGLL**) triggered the biggest response by patient HH-03 and *in vitro* binding data as well as preliminary restriction experiments suggest that this epitope was likely restricted by DRB1^*^04 (Supplementary Figure 7) in this patient. However, restriction by other molecules like DP and DQ molecules were not tested and cannot be excluded as alternative but less likely restricting molecules.

For both EXP1-P15 and EXP1-P20 we also tested variants found on UniProt (32). EXP1-P15 variant 2 contained serine (S) instead of alanine (A) at position 13 and showed the biggest response. EXP1-P20 variant 1 contained arginine (R) instead of histidine (H) at position 14 and EXP1-P20 variant 2 contained arginine (R) instead of lysine (K) at position 11. EXP1-P20 variant 2 triggered a much bigger response than variant 1. These results show that the change of a single amino acid can lead to an improvement of binding and therefore recognition and are in line with prior publications (54). No variants for EXP1-P13 could be found in online databases (Supplementary Table 1).

## Discussion

Here, we present an immunological study of the *P. falciparum* EXP1-specific T cell response on a single epitope level using PBMCs of a large and well-characterized patient cohort. In concordance with the results of previous studies, frequencies of *ex vivo* EXP1-specific T cell responses were low. However, with our sensitive *in vitro* testing approach we detected T cell responses in 21 of the 45 patients, and discovered responses directed against 15 of 31 peptides (48.4%) spanning the entire EXP1 protein, making this the first study to comprehensively describe responses against a broad range of EXP1-specific epitopes. For most *P. falciparum* antigens previous studies only studied vaccine-primed T cell responses in malaria-naïve volunteers within human vaccine trials (25, 55) which cannot be directly compared with *P. falciparum* T cell responses generated during a natural malaria infection. Other studies used small and partly HLA-A2 pre-selected study populations (54). HLA-A2 is the most common expressed class I HLA in Caucasians, but not as commonly expressed in African individuals. As a result, these previous studies have limited applicability for vaccine design and immunomonitoring in malaria endemic countries. Other studies used *in silico* predicted epitopes or peptide pools instead of comprehensive, overlapping peptide sets (24, 56, 57). However, this approach does not allow the identification of single epitopes and immunogenic parts of antigens. Published *P. falciparum* specific CD4+ and CD8+ T cell epitopes as well as HLA-restriction (if known) and newly detected CD4+ T cell epitopes from this study are shown in Supplementary Figure 1. We also assessed important clinical parameters such as the HLA type, origin and parasitaemia in our study cohort, but no significant correlation with the CD4+ T cell response could be shown.

Of note, EXP1-P15 (aa70-85) and EXP1-P13 (aa60-74) elicited a CD4+ T cell response in a quarter of our study group that had a diverse HLA-background. The minimal length was experimentally narrowed down in two patients and the most likely restricting HLA-molecule of each epitope was evaluated using our *in vitro* HLA class II binding data (Table 3), *in silico* predictions (Supplementary Table 2A) and comparing it with the HLA molecules of responding patients (Supplementary Table 2B). EXP1-P15 is likely to be restricted by HLA-DRB1^*^01:01, DRB1^*^04:01, DRB1^*^04:02, DRB1^*^04:05, DRB1^*^07:01, DRB1^*^10:01, DRB1^*^13:03, DRB1^*^15:02, and DRB1^*^15:03. Interestingly, six patients in our cohort also expressed DRB1^*^07:01 but did not respond to EXP1-P15. Heterology of the protein sequence of the autologous infecting strain, or slightly differing immunogenic epitope cores between different patients with the same HLA molecules (29) could explain, why some patients were able to recognize a certain epitope unlike others even though they express the same HLA. In this study we have only tested the binding to DRB-molecules, since they restrict the majority of class II responses (~50%), but acknowledge that restrictions by HLA-DQ and DP molecules are also possible (58). One study examining the HLA-DP, DQ, and DRB3/4/5 restricted responses showed that Dengue virus-specific CD4+ T cell responses were of lower magnitude compared to HLA-DRB1 (58, 59). This is consistent with their lower levels of expression (58). Future studies should experimentally confirm the HLA-restriction of these EXP1 T cell epitopes and possibly evaluate restriction by HLA-DP or DQ molecules.

Further detailed *in vitro* HLA-restriction and peptide truncation experiments to establish optimal peptide length and HLA-restriction (i.e., with single HLA-molecule transfected cell lines or by blockade with anti-DR, and-DQ or anti-DP antibodies) of peptide specificities defined in this study need to be conducted in future follow-up studies.

We still might underestimate the breadth of the *P. falciparum* EXP1-specific T cell response. In this study design we aimed to expand and define dominant peptide-specific T cell responses producing IFNγ to define the breadth of the CD4+ T cell repertoire after short term cell cultures and using exogenous IL-2 which is known to create a type 1 T helper (Th1) cell cytokine shift (60) and no statement can be made about the original *ex vivo* phenotype or functionality of these cells. Future studies should therefore further characterize T cell responses by directly assessing *ex vivo* secretion (e.g., with ELISPOT) of different, additional cytokines (e.g., IL-4, IL-10) that potentially could have been missed using the current approach.

Further subdominant responses might be detectable using peptide sets of peptide variants matching autologous sequences or by applying more sensitive, but far more labor-intensive techniques, like single cell dilution cloning. Patient HH-21 was infected with *P. vivax* and not with *P. falciparum* but the EXP1-specific cultures nevertheless elicited a CD4+ T cell response against EXP1-P15 indicating that immunity against malaria can indeed be cross-species specific. Of note, the sequence of EXP1-P15 (aa70-85) is well-conserved (Supplementary Table 1), potentially making this epitope an interesting candidate for immunomonitoring by MHC-multimer technology or an epitope to be used for a suitable subunit vaccine.

Also, our analysis is limited to samples drawn directly after hospital admission during acute malaria and it is not known how long each patient had been infected. Of note, if we stratify our data according to patients with and without a response, surprisingly the time after malaria treatment did not significantly differ between either group at this early phase of infection (Supplementary Figure 6B). The single cases for which we were able to analyze the T cell response at later time points would let us hypothesize that the naturally primed EXP1 specific T cell response rapidly wanes over the weeks after successful treatment.

Although EXP1 is likely to be essential for the development and the survival of the parasite, its exact function in the *P. falciparum* malaria life cycle is far from clear and more investigation as well as linkage between important and conserved regions and immunopotent epitopes are necessary (61). Since EXP1 is expressed at the liver stage during a malaria infection we also expected to detect CD8+ T cell responses. However, all CD8+ T cell responses detected in this study were weak and coincided with a parallel CD4+ T cell response directed against the same peptide in the same patient and we did not follow-up on fine-mapping these subdominant T cell responses (Supplementary Figure 4). It has to be considered that the design of our overlapping peptide set, with an average length of 15 amino acids, is biased toward detection of CD4+ T cell responses and could therefore explain low CD8+ T cell responses. Furthermore, prior publications showed that the blood phase of a malaria infection suppresses immune cells of the liver stage (62, 63). This could also explain the lack of CD8+ T cell responses in our cohort since all patients had an extensive blood phase with strong symptoms and the need to be hospitalized. It has been discussed that CD4+ T cells might play an important role in priming other immune cells (i.e., NK cells) and in evoking a strong antibody response which is necessary for immune control (64, 65). If an efficient induction of immunity against *plasmodial* infection by CD8+ T cells depends on CD4+ T cells, is therefore an important question to address in the development of an effective vaccine.

EXP1 is also called circumsporozoite-related antigen due to a similarity between the amino acid sequence of EXP1 and the circumsporozoite protein (CSP). The CSP is by far the most investigated malaria antigen and it is also part of the RTS,S vaccine trial (66). The amino acid sequence **NANPDADSESNGEPN** of EXP1 is similar to the NANP-repeat region of the CSP which has so far only shown few T cell responses (67, 68). Interestingly, we detected T cell responses against EXP1-P25 which is located in this NANP-related region of EXP1 and future studies are necessary to evaluate if not only CD4+ T cell and antibody responses against the NANP-repeat region but also CD8+ T cell responses can be found. The cross reactivity of vaccine-induced CSP and EXP1-primed T cell responses should be tested in future studies.

The RTS,S vaccine only showed short lived efficacy and also naturally acquired immunity only lasts a few months (69). Epidemiological evidence described that antibodies to *Plasmodium* antigens are inefficiently generated and rapidly lost without continued parasite exposure (70). We also investigated longitudinal follow-up samples of a subset of patients and could show that the EXP1-specific T cell response primed by natural infection waned over time and was not detectable 12 months after the diagnosis and therapy of acute malaria (data not shown). Unfortunately, it was difficult in the present study to collect samples at later timepoints after the patients were discharged. And future, well-structured and prospective longitudinal studies that describe the dynamics of the priming and the breadth, magnitude and quality of the T cell response directed against different malaria antigens are necessary.

This current study aimed to primarily discover novel *P. falciparum*-specific epitopes with a frequency high enough to be detectable e.g., after enrichment by MHC class II multimer technology. This technology will allow the characterization of the phenotype of T cells which could help to understand the role but also the complications of T cells during malaria infections. In previous studies, EXP1 has shown to generate a strong antibody response in naturally exposed individuals and high antibody titers specific to EXP1 aa73-162 correlated with a high level of IL-6 production to the same peptide (20). This strong recognition by B and T cells suggest that the whole sequence of EXP1 may represent a suitable malaria antigen for a subunit vaccine construct (20).

In summary, a broad range of *P. falciparum* EXP1-specific CD4+ T cell responses can be detected after *in vitro* expansion in nearly half of our patients of different origin and within a diverse HLA molecule background. We did not find any correlation of the number of T cell responses and relevant clinical parameters in this cohort. These detailed data on *P. falciparum* EXP1-specific T cell epitopes will be helpful for the development of tools like MHC class II multimers or to monitor the immune response on an epitope level during future malaria vaccine trials.

## Data Availability Statement

The datasets generated for this study are available on request to the corresponding author.

## Ethics Statement

All subjects gave written informed consent in accordance with the Declaration of Helsinki and the study was approved by the local ethics board of the Ärztekammer Hamburg (PV 4238).

## Author Contributions

JH, TJ, and JSzW: conception. JH and JSzW: first draft. MM performed the HLA-typing. JS: the *in vitro* binding restrictions. All authors: important contributions and proofreading.

### Conflict of Interest

The authors declare that the research was conducted in the absence of any commercial or financial relationships that could be construed as a potential conflict of interest.

## Supplementary Material

Supplementary Table 1 13 different sequences of *P. falciparum* EXP1 and two sequences of *P. vivax* EXP1. We found 13 different sequences for *P. falciparum* EXP1 in the online database UniProt (31). The sequence of *P. vivax* EXP1 that resembles *P. falciparum* EXP1-P15 is framed. Some sequences are only fragments and therefore shorter than 162 amino acids.

Supplementary Table 2 *In silico* **(A)** and most likely **(B)** binding predictions for EXP1 peptides that elicited a CD4+ T cell response. **(A)** *In silico* binding predictions for HLA-DRB1 molecules. *In silico* binding predictions were performed as described for 15 EXP1 peptides that at least one patient's CD4+ T cells responded to. The threshold for the binding affinity was defined as 5,0; the most probable binders are displayed in bold. The MHC class II *in silico* binding predictions were made using the IEDB analysis resource consensus tool (38–40) combining predictions from ANN (41, 42) and SMM (43). **(B)** Most likely HLA-restrictions of the EXP1 peptides that elicited a response as determined by *in silico* and *in vitro* predictions matching the patients' HLA molecules.

Supplementary Figure 1 Epitope map of EXP1. EXP1 is a 162 amino acid long protein consisting of a signal sequence (aa1–23), an N-terminus (aa23–79), a transmembrane domain (aa79–101), and a C-terminus (aa101–162) (71). Already published CD4+ and CD8+ T cell epitopes as well as HLA-restriction (if known) and newly detected CD4+ T cell epitopes from this study are marked within this epitope map. Marked in light gray: Published CD8+ T cell epitopes: **KILSVFFLA** (23, 25)**, ALFFIIFNK** (23)**, ATSVLAGL** (21)**, VLAGLLGNV** (23)**, GLLGNVSTV** (23, 25)**, VLLGGVGLVL** (9, 23). Marked in gray: Published CD4+ T cell epitopes: **KSKYKLATSVLAGLL** (22, 25), **YKLATSVLAGLLGVVSTVLLGG** (24, 25), **AGLLG(V/N)VSTVLLGGV** (25), **GLVLYNTEKGRHPFKIGSSD** (9, 24). Marked in dark gray: CD4+ T cell epitopes that elicited a CD4+ T cell response in the current study.

Supplementary Figure 2 Exemplary gating strategy. Dead cells, CD14+ cells and CD19+ cells were excluded. After gating on single cells and lymphocytes, CD3+ cells were selected. Subsequent IFNγ response was based on either CD8+ or CD4+ T cells.

Supplementary Figure 3 Countries that were visited by 41 malaria patients are marked by a black dot. Most patients who were treated for malaria at University Hospital of Hamburg had traveled and returned from Western Africa: nine patients had traveled to Nigeria, eight to Ghana, and five patients to Togo.

Supplementary Figure 4 Exemplary ICS dot blot of a *P. falciparum-specific* CD8+ T cell response of HH-45 against EXP1-P15. **(A)** CD8+ T cells are gated with a negative control (left) and a positive peptide response (right) against EXP1-P15 (aa70-85) by HH-45. DMSO and R10-medium were added to the negative control. **(B)** *P. falciparum-specific* CD4+ T cell response: black, *P. falciparum-specific* CD8+ T cell response: gray. Patient HH-16, HH-18, HH-20 and HH-25 showed a CD8+ T cell response that coincided with a CD4+ T cell response against EXP1-P02, P07, P09, P13, and P15. Interestingly, we did not detect a CD8+ response against a peptide without a CD4+ response against the same peptide.

Supplementary Figure 5 Breadth of the P. falciparum-specific T cell response in correlation with age [years] and sex. **(A)** No CD4+ or CD4+ T cell response compared to age. **(B)** No CD4+ or CD4+ T cell response compared to sex. No correlation with the CD4+ T cell response compared with age or sex could be shown.

Supplementary Figure 6 Breadth of the *P. falciparum*-specific T cell response in correlation with patient characteristics. **(A)** Number of *P. falciparum*-specific T cell responses detected in patients from Germany and in patients from the African continent. **(B)** Correlation of the *P. falciparum*-specific T cell response with the days since the start of the malaria therapy. **(C)** Correlation of the *P. falciparum*-specific T cell response compared to a complicated or uncomplicated course of the malaria infection. **(D)** Number of *P. falciparum*-specific T cell responses detected in patients with the first malaria infection and patients with prior malaria infection.

Supplementary Figure 7 Restriction assay for EXP1-P15 with PBMCs of patient HH-03 (DRB1^*^04:05P,^*^15:02P, DQB1^*^04:02P, ^*^05:02P). This figure shows the results of the restriction assay for EXP1-P15. PBMCs of patient HH-03 (DRB1^*^04:05P,^*^15:02P, DQB1^*^04:02P, ^*^05:02P) were cultivated for 14 days with EXP1-P15 (concentration 10 μg/ml). We then used PBMCs of matched donors as antigen presenting cells (APCs). These APCs were incubated with EXP1-P15 (concentration 10 μg/ml) for 20 minutes, then washed six times and used to stimulate the PBMCs of patient HH-03. Donor 1 (DRB1^*^15:02, ^*^11:01; DQB1^*^03:01P, ^*^06:01P) matched the HLA molecule HLA-DRB1^*^15:02 of patient HH-03 and Donor 2 (DRB1^*^04:01P,^*^04:04P; DQB1^*^03:02P) the HLA allele HLA-DRB1^*^04. The results show that HLA-DRB1^*^04 most likely presents EXP1-P15. As negative control PBMCs of Donor 1 and 2 that were not incubated with EXP1-P15 were used to stimulate PBMCs of patient HH-03. As positive control we used PBMCs of patient HH-03 incubated with EXP1-P15 for 20 min and washed for six times. Donor 1 and 2 expressed different HLA-DQB1 molecules than patient HH-03 and we could therefore exclude the possibility of presentation by DQB1. Unfortunately, HLA-DQA1 alleles were not assessed for Donor 1 and 2 and presentation by these molecules could therefore not be excluded and could explain, why the positive control (frequency: 9.51%) showed a higher frequency than the APCs of Donor 2 (frequency: 2.35%).
