# Supplementary material for: Detection of EXP1-Specific CD4+ T Cell Responses Directed Against a Broad Range of Epitopes Including Two Promiscuous MHC Class II Binders During Acute Plasmodium falciparum Malaria
Source: Front Immunol. 2020 Jan 22;10:3037. doi: 10.3389/fimmu.2019.03037 (PMC6993587; doi:10.3389/fimmu.2019.03037)
Supplement: Supplementary Table 1 — 13 different sequences of P. falciparum EXP1 and two sequences of P. vivax EXP1. We found 13 different sequences for P. falciparum EXP1 in the online database UniProt (31). The sequence of P. vivax EXP1 that resembles P. falciparum EXP1-P15 is framed. Some sequences are only fragments and therefore shorter than 162 amino acids. [file Presentation_1.pptx]

## Slide 1
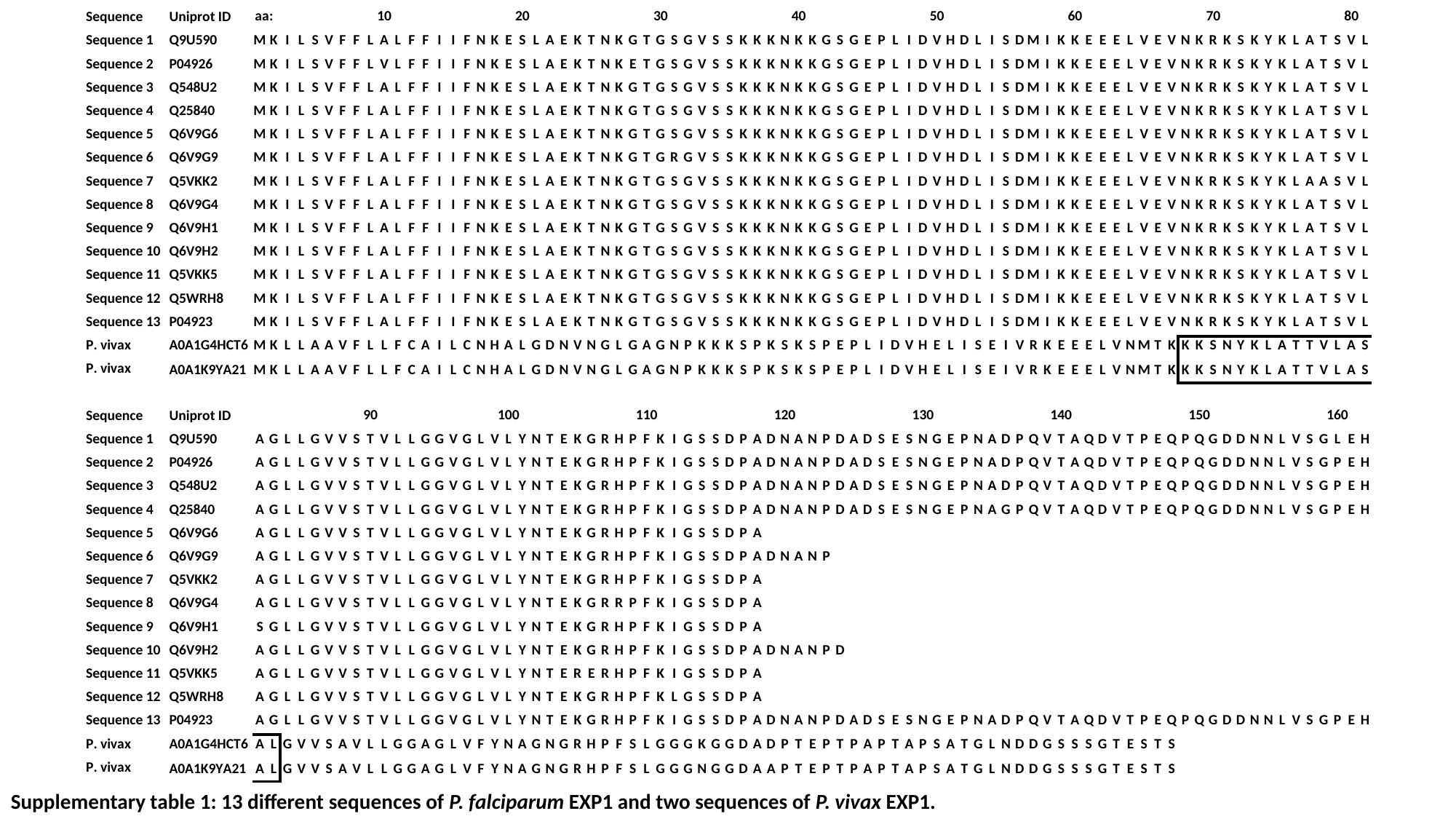

Supplementary table 1: 13 different sequences of P. falciparum EXP1 and two sequences of P. vivax EXP1.

## Slide 2
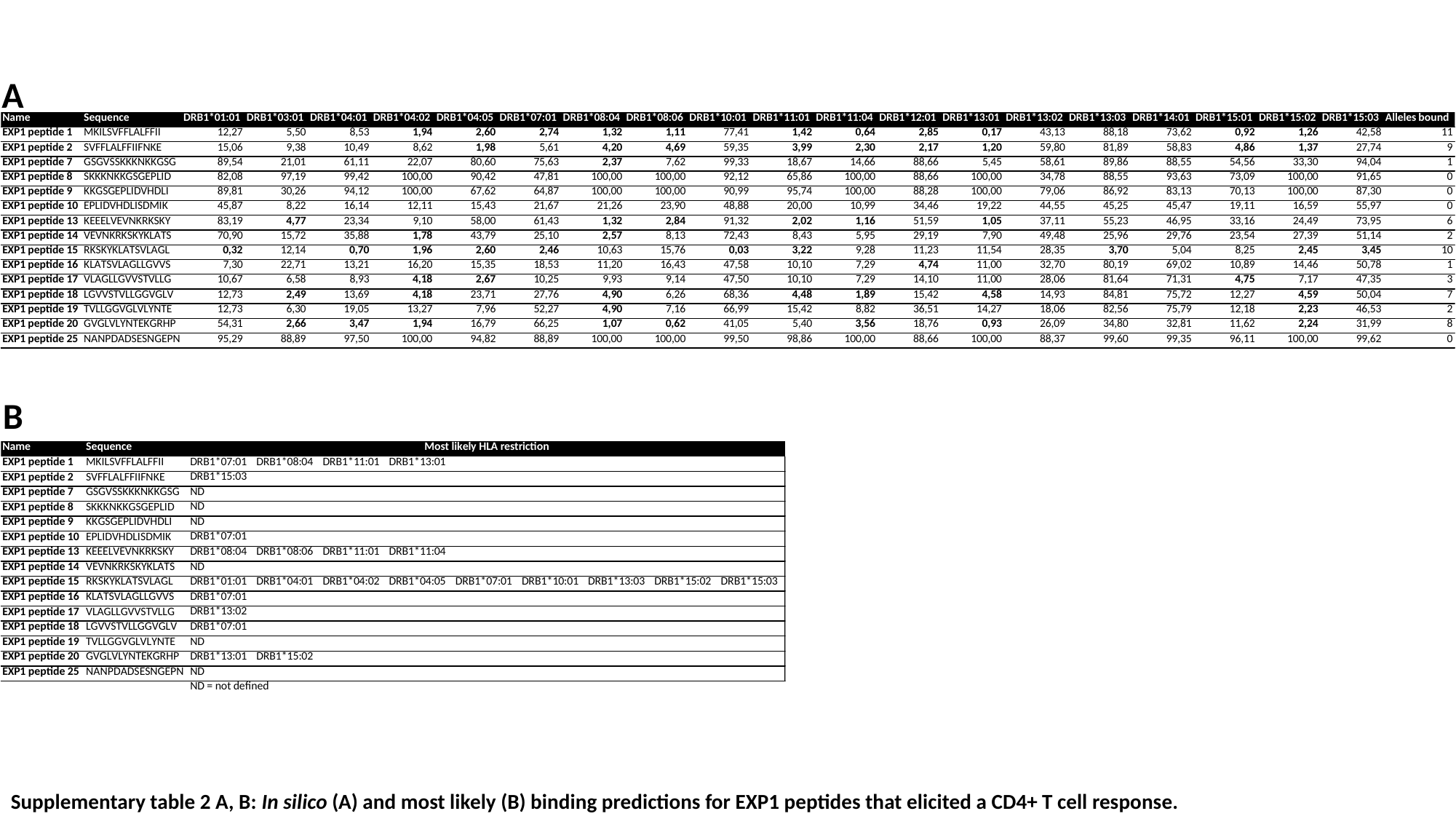

A
B
Supplementary table 2 A, B: In silico (A) and most likely (B) binding predictions for EXP1 peptides that elicited a CD4+ T cell response.

## Slide 3
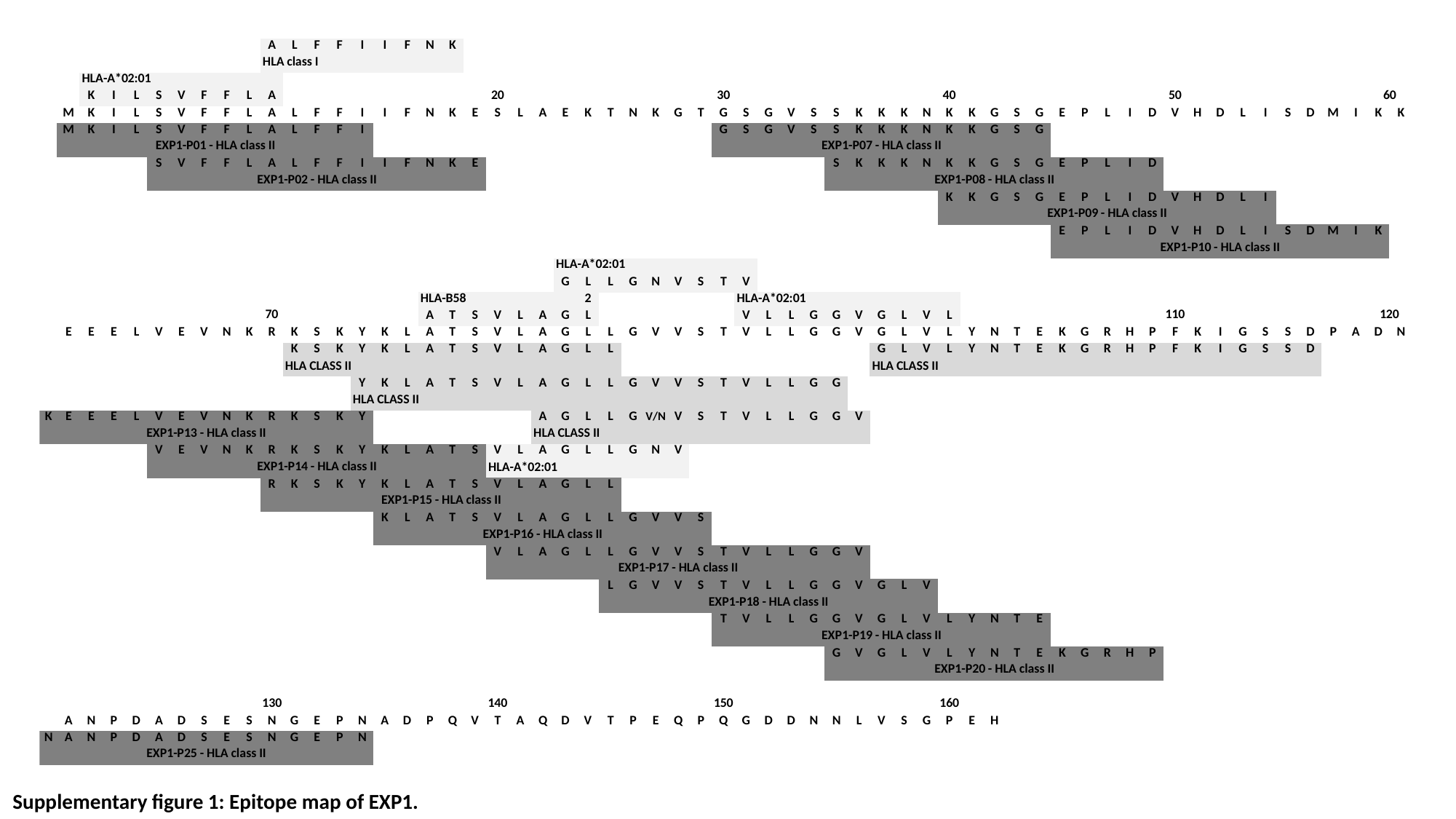

Supplementary figure 1: Epitope map of EXP1.

## Slide 4
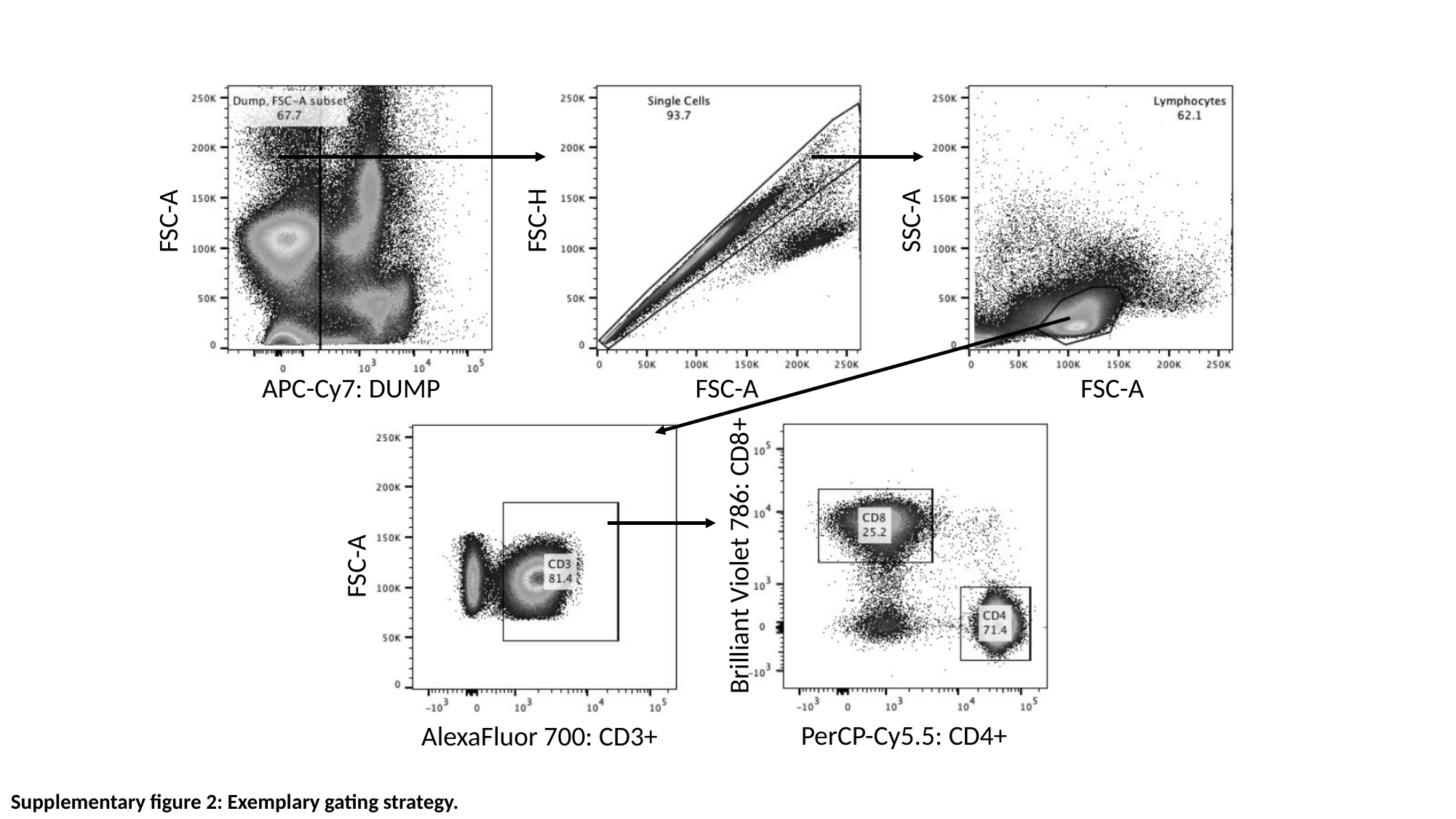

FSC-H
FSC-A
SSC-A
APC-Cy7: DUMP
FSC-A
FSC-A
Brilliant Violet 786: CD8+
FSC-A
PerCP-Cy5.5: CD4+
AlexaFluor 700: CD3+
Supplementary figure 2: Exemplary gating strategy.

## Slide 5
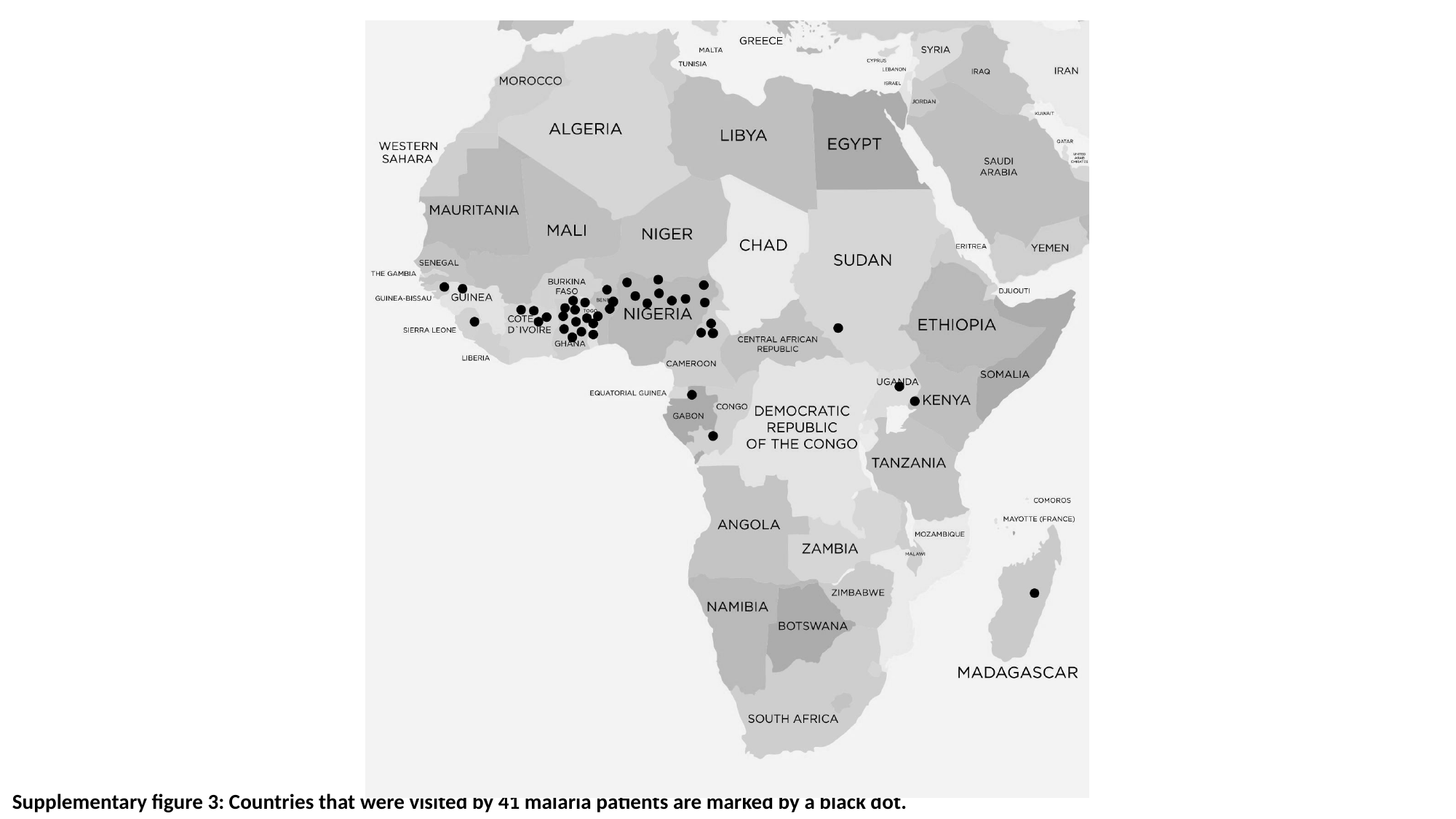

Supplementary figure 3: Countries that were visited by 41 malaria patients are marked by a black dot.

## Slide 6
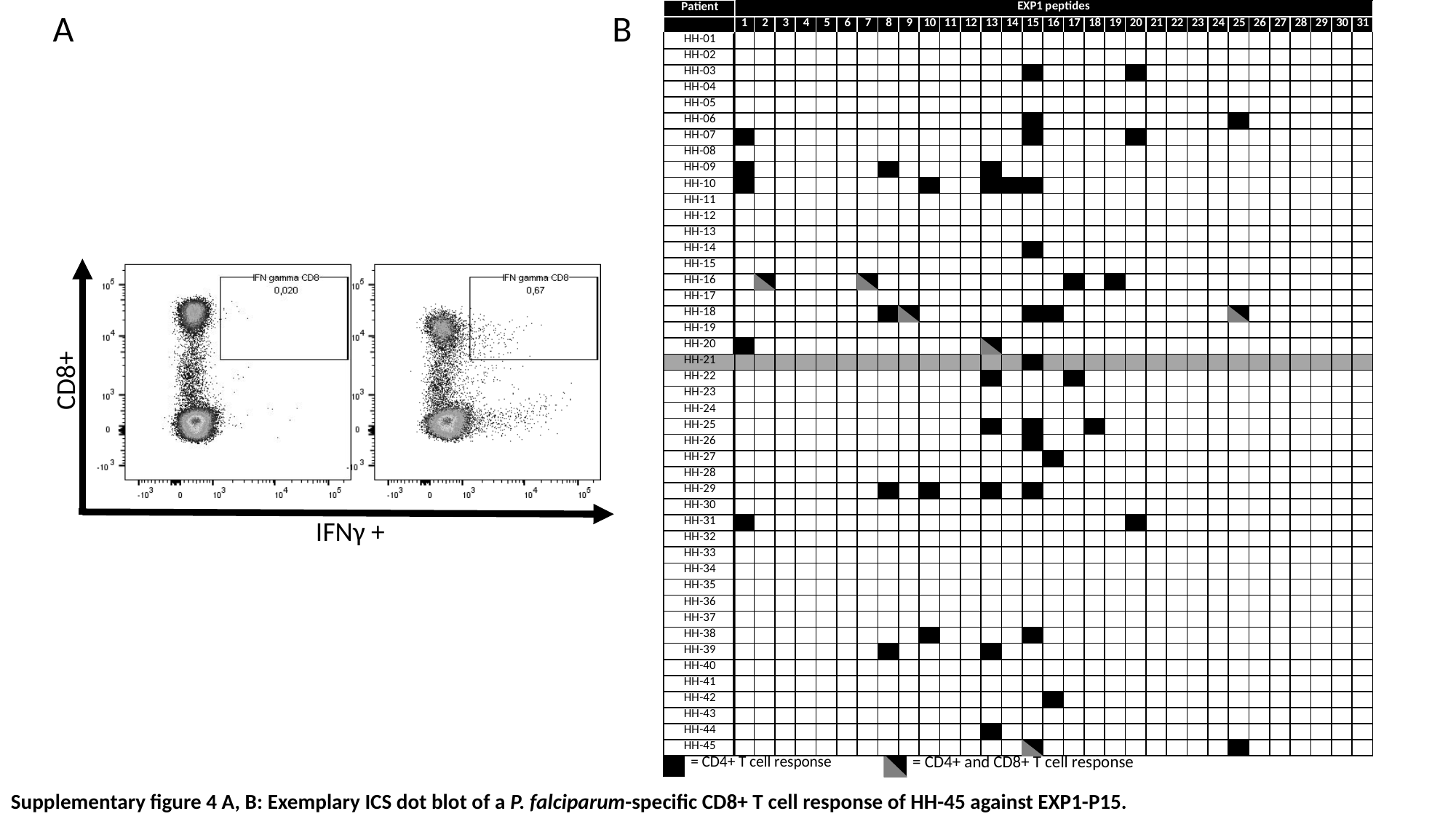

B
A
CD8+
IFNγ +
Supplementary figure 4 A, B: Exemplary ICS dot blot of a P. falciparum-specific CD8+ T cell response of HH-45 against EXP1-P15.

## Slide 7
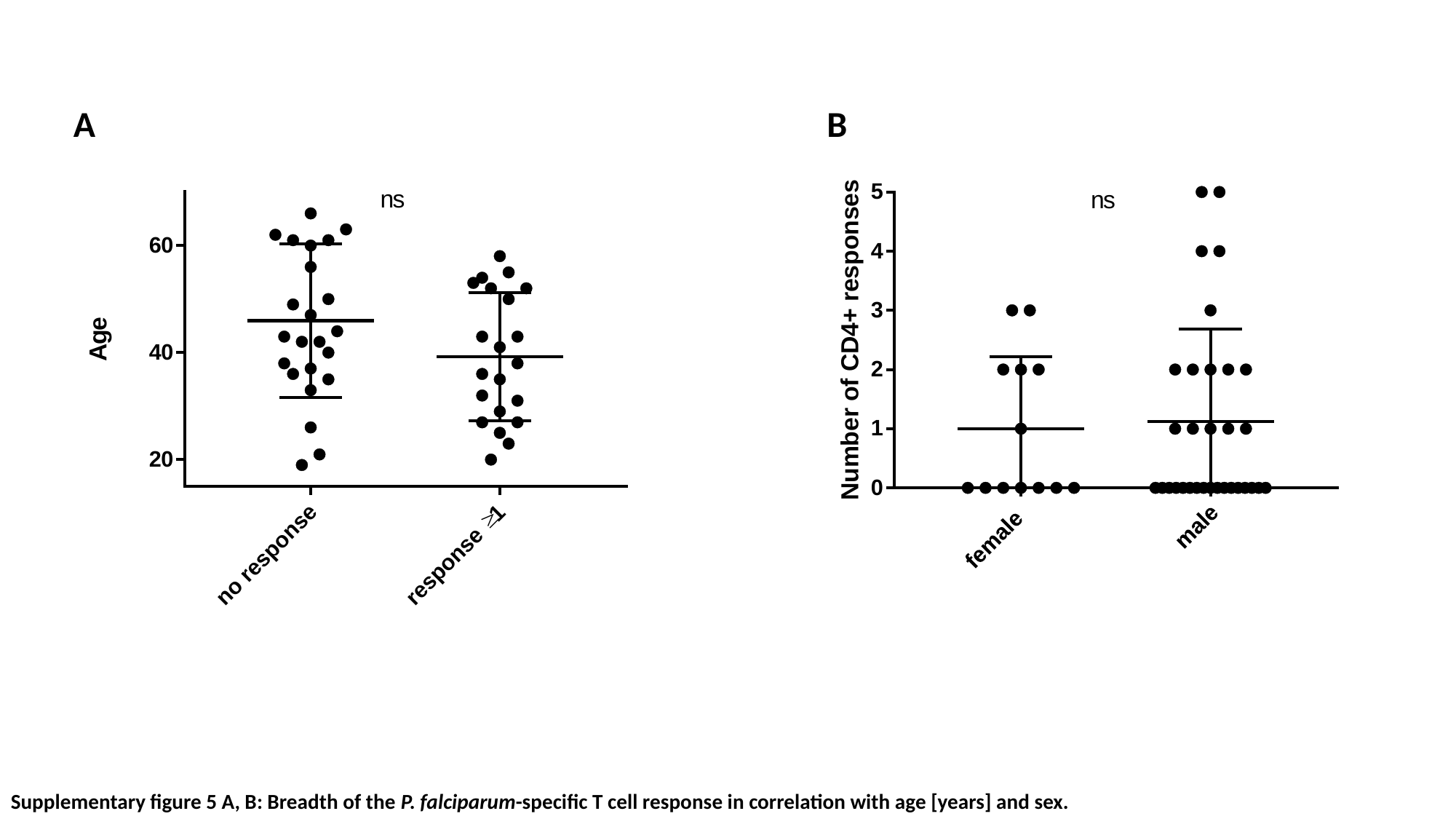

A
B
Supplementary figure 5 A, B: Breadth of the P. falciparum-specific T cell response in correlation with age [years] and sex.

## Slide 8
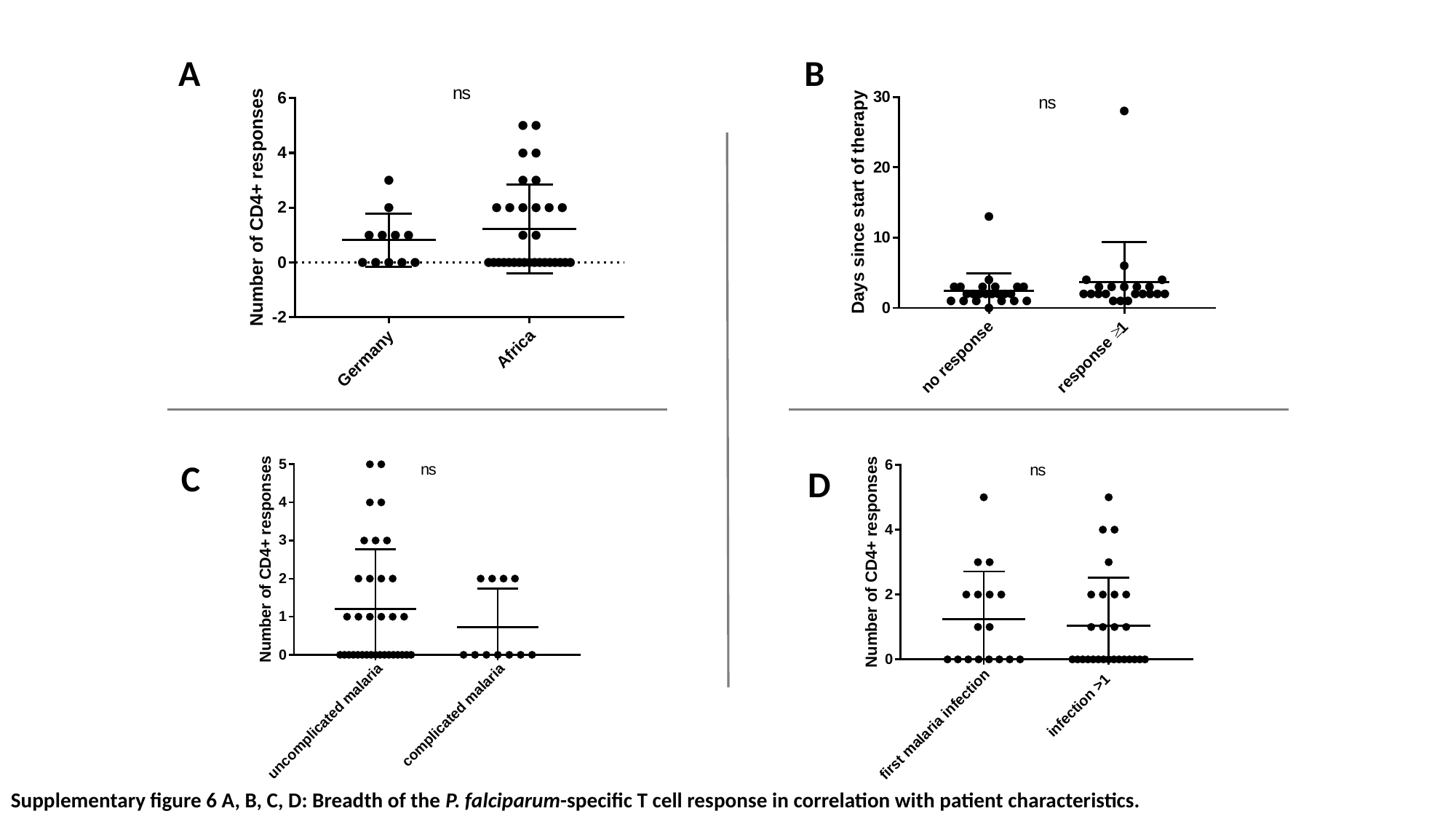

A
B
C
D
Supplementary figure 6 A, B, C, D: Breadth of the P. falciparum-specific T cell response in correlation with patient characteristics.

## Slide 9
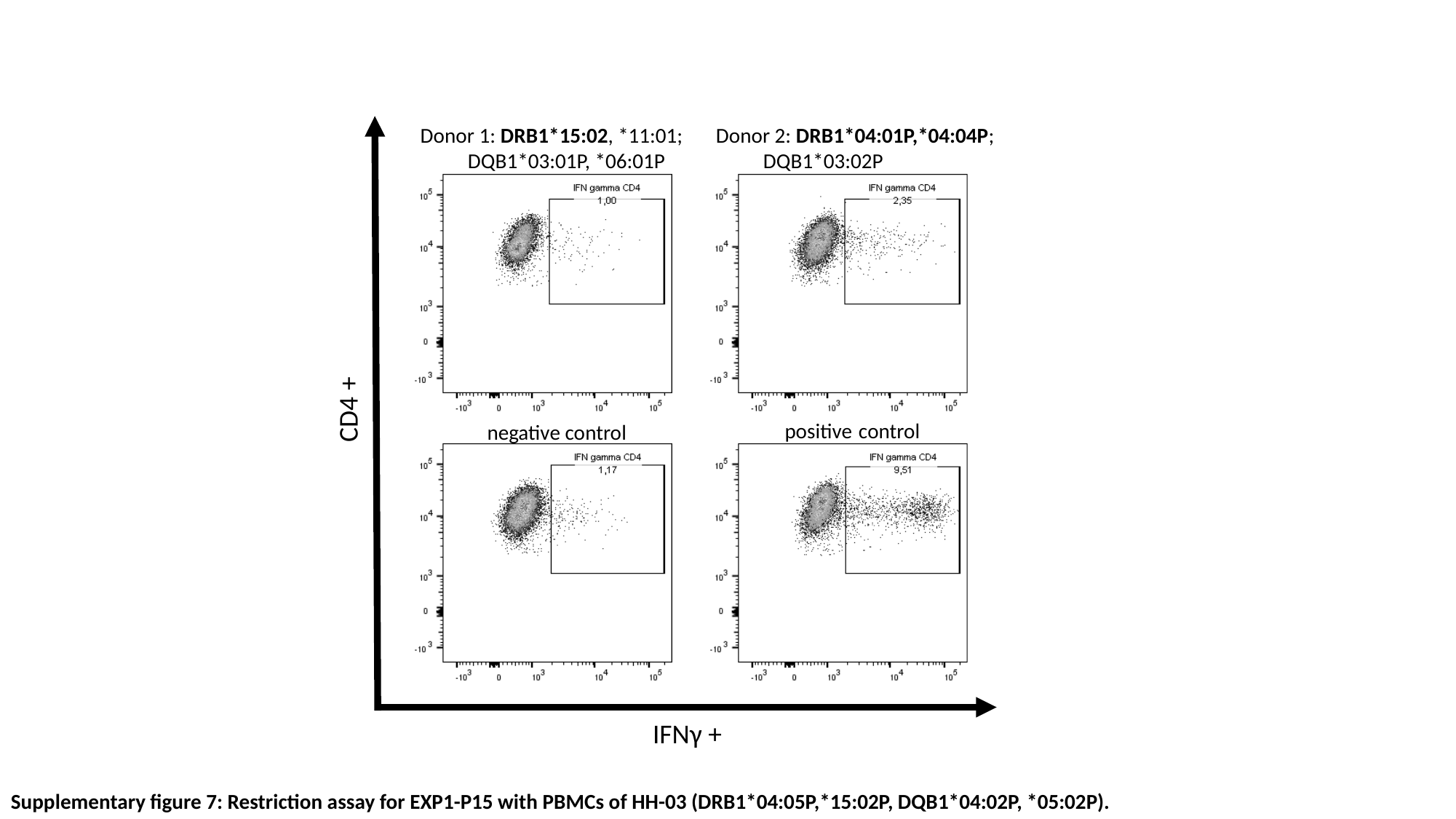

Donor 1: DRB1*15:02, *11:01;
 DQB1*03:01P, *06:01P
Donor 2: DRB1*04:01P,*04:04P;
 DQB1*03:02P
CD4 +
IFNγ +
positive control
negative control
Supplementary figure 7: Restriction assay for EXP1-P15 with PBMCs of HH-03 (DRB1*04:05P,*15:02P, DQB1*04:02P, *05:02P).
